# Evaluation of preoperative diagnostic methods for resectable pancreatic cancer: a diagnostic capability and impact on the prognosis of endoscopic ultrasound-guided fine needle aspiration

**DOI:** 10.1186/s12876-021-01955-7

**Published:** 2021-10-18

**Authors:** Akinori Maruta, Takuji Iwashita, Kensaku Yoshida, Shinya Uemura, Ichiro Yasuda, Masahito Shimizu

**Affiliations:** 1 First Department of Internal Medicine, Gifu University Hospital, 1-1 Yanagido, Gifu, 501-1194 Japan; 2 Third Department of Internal Medicine, Toyama University Hospital, Toyama, Japan

**Keywords:** Pancreatic cancer, Preoperative, EUS-FNA, ERCP, FNB

## Abstract

**Background:**

A pathological diagnosis of pancreatic cancer should be performed as much as possible to determine the appropriate treatment strategy, but priorities and algorithms for diagnostic methods have not yet been established. In recent years, the endoscopic ultrasound-guided fine-needle aspiration (EUS-FNA) has become the primary method of collecting tissues from pancreatic disease, but the effect of EUS-FNA on surgical results and prognosis has not been clarified.

**Aims:**

To evaluate the diagnostic ability of EUS-FNA and its effect on the preoperative diagnosis, surgical outcome, and prognosis of pancreatic cancer.

**Methods:**

Between January 2005 and June 2017, 293 patients who underwent surgical resection for pancreatic cancer were retrospectively evaluated. The outcomes of interest were the diagnostic ability of EUS-FNA and its influence on the surgical results and prognosis.

**Results:**

The diagnostic sensitivity of EUS-FNA was 94.4%, which was significantly higher than that of endoscopic retrograde cholangiopancreatography (ERCP) (45.5%) (*p* < 0.001). The adverse event rate in ERCP was 10.2%, which was significantly higher than EUS-FNA (1.3%) (*p* = 0.001). Patients were divided into FNA group (N = 160) and non-FNA group (N = 133) for each preoperative diagnostic method. In the study of surgical curability R0 between the two groups, there was no significant difference in FNA group (65.0% [104/160]) and non-FNA group (64.7% [86/133], *p* = 1.000). In the prognostic study, 256 patients with curative R0 or R1 had a recurrence rate was 54.3% (70/129) in the FNA group and 57.4% (73/127) in the non-FNA group. Moreover peritoneal dissemination occurred in 34.3% (24/70) in the FNA group and in 21.9% (16/73) in the non-FNA group, neither of which showed a significant difference. The median survival times of the FNA and non-FNA groups were 955 days and 799 days, respectively, and there was no significant difference between the two groups (log-rank *p* = 0.735). In the Cox proportional hazards model, factors influencing prognosis, staging, curability, and adjuvant chemotherapy were the dominant factors, but the preoperative diagnostic method (EUS-FNA) itself was not.

**Conclusions:**

EUS-FNA is a safe procedure with a high diagnostic ability for the preoperative examination of pancreatic cancer. It was considered the first choice without the influence of surgical curability, postoperative recurrence, peritoneal dissemination and prognosis.

## Background

Pancreatic cancer is the fourth leading cause of cancer-related deaths in the United States, with 227,000 deaths per year worldwide [1, 2]. Surgical resection is strongly recommended for resectable pancreatic cancer as it is the only method that results in a complete cure. However, the surgical resection of the pancreas is associated with certain rates of adverse events due to invasive nature of the surgery itself. Therefore, accurate preoperative differential diagnosis and staging are very important to avoid unnecessary surgery. It is also recommended to diagnose pancreatic cancer based on pathological analysis as much as possible, although priorities and algorithms for diagnostic methods have not yet been established.

Endoscopic ultrasound-guided fine-needle aspiration (EUS-FNA) was first reported by Vilmann et al. [3] in 1992 and has been increasingly used worldwide to obtain pathological samples from pancreatic tumors. The diagnostic capabilities for malignancy of EUS-FNA were considered to be very high for pancreatic cancer, which was reported to have a sensitivity of 0.85–0.89 and specificity of 0.96–0.98 in meta-analyses [4–6]. The adverse event rate of EUS-FNA was reported to be 1.7% [7]. Therefore, it is considered a safe procedure. However, the indications of EUS-FNA for pancreatic tumors prior to surgery have remained controversial because of concerns regarding needle-track seeding and tumor dissemination. Some reports have suggested that the use of EUS-FNA neither increases the risk of peritoneal recurrence nor influences recurrence-free survival or overall survival [8–10]. Despite these, there have been several cases of needle-track seeding at the gastric wall, which was most likely caused by EUS-FNA [11–15]. The purpose of this study was to examine the diagnostic ability of EUS-FNA and the influence on the surgical results and prognosis in patients with pancreatic cancer.

## Patients and methods

### Patients

This was a multicenter, retrospective cohort study conducted at one academic and two tertiary care centres. The analysis included data on all patients who underwent surgical resection for pancreatic cancer between January 2005 and June 2017. However, patients who met the following criteria were excluded from the analysis: previous history of upper intestinal surgery or any type of malignancy 5 years after the surgery. Written informed consent for endoscopic procedures was obtained from all patients. The consent for participation of patients in this study and its publication was obtained through an opt-out methodology. The study was conducted in accordance with the principles of the Declaration of Helsinki. The study protocol was approved by the Institutional Review Board at each institution and was registered in the UMIN Clinical Trials Registry (UMIN000025993).

### Definitions

The patients were divided into two groups; 160 patients who underwent preoperative EUS-FNA (FNA group) and 133 patients who did not (non-FNA group). Survival time was calculated from the day of surgery to the day of death. Operative curability was defined as follows. R0, no evidence of residual tumor; R1, residual tumor in pathological analysis; R2, residual tumor macroscopically. The staging was based on the General Rules for the Study of Pancreatic Cancer (6^th^ Edition, Revised Version) of the Japan Pancreas Society. Adverse Events of the endoscopic procedures were defined according to the lexicon for endoscopic adverse events by the American Society of Gastrointestinal Endoscopy [16].

### Endoscopic procedures

All endoscopic procedures were performed by well-experienced endoscopists using both EUS- and ERCP-related procedures. EUS-FNA was performed under conscious sedation with intravenous administration of midazolam and pentazocine. A convex-type echoendoscope (GF-UC240P-AL5, or GF-UCT260; Olympus, Tokyo, Japan) was used for the examinations. In EUS-FNA, the pancreatic lesion was first visualized by EUS, then the needle was advanced into the lesion through the gastric or duodenal wall. For transgastric punctures, a 19G needle was actively used, and for transduodenal punctures, finer needles, such as 22G and 25G, were often used. After removal of the stylet, a syringe was attached to the needle to apply negative pressure (10 ml) followed by to-and-fro movement several times. The specimen was evaluated macroscopically (macroscopic on-site evaluation; MOSE), as we have previously reported [17]. If it was insufficient in the first puncture, the suction pressure in the second puncture was increased to 15-20 ml, and if there was a lot of blood contamination, the slow pull method was utilized. The needle was then pulled back and removed using EUS. The specimen obtained by FNA was spelled out on a glass slide through the reinsertion of the stylet into the needle. The whitish-visible specimen was placed into a formalin bottle for histological analysis. The smear was made with the remaining specimen on a glass slide and fixed with pure alcohol for cytological analysis.

### Data analysis

The primary outcome of this study was to evaluate the influence of EUS-FNA on the surgical outcomes and prognosis of pancreatic cancer. The secondary outcomes were the diagnostic capability of EUS-FNA for pancreatic tumors and the adverse event rates from endoscopic procedures. Comparisons were performed using Fisher’s exact test or Pearson’s chi-squared test, as appropriate, for categorical variables, and the Mann– Whitney U-test was performed for continuous variables. The Kaplan–Meier method was performed to estimate survival time, and the log-rank test was used for comparing the two groups. A Cox proportional-hazards model was used to estimate hazard ratios for prognosis, and the possible risk factors associated with survival time were also assessed. The following variables were considered to be candidate risk factors: preoperative diagnostic methods (FNA or non-FNA), age, sex, tumor location, tumor diameter, clinical stage, operation curability, and adjuvant chemotherapy. In the analyses, continuous variables were transformed into dichotomous variable with a cut-off value of the median number. The factors with a *p*-value of < 0.20 in the univariate analyses and the diagnostic method (EUS-FNA) were further assessed in the multivariate analysis. A two-sided *p*-value of < 0.05 was considered to be statistically significant. Statistical analyses were performed using JMP version 10 (SAS Institute, Inc., Cary, NC, USA) or R ver. 3.3.1 (R Foundation for Statistical Computing, Vienna, Austria; http://www.R-project.org/).

## Results

### Basic characteristics

**Fig. 1:**
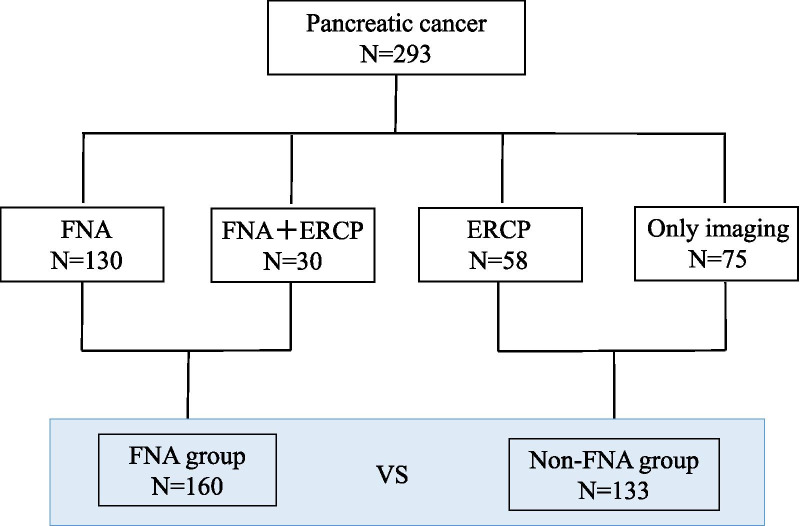
Patient flow of this study. FNA, fine needle aspiration; ERCP, endoscopic retrograde chorangiopancreatography

A total, 293 patients were enrolled this study. The preoperative diagnostic methods of pancreatic cancer included EUS-FNA in 130 patients, ERCP in 58 patients and both EUS-FNA and ERCP in 30 patients. In the remaining 75 patients, pancreatic cancer was diagnosed based only on imaging findings. Furthermore, we classified the patients into the FNA and non-FNA groups. The patient’s breakdown is shown in Fig. 1. The patient characteristics are summarized in Table 1. There was no significant difference between the FNA and non-FNA groups, except for sex. There were significantly more males in the FNA group.

**Table 1:** Baseline characteristics of patients with solid pancreatic tumor

	FNA group (N = 160)	Non-FNA group (N = 133)	*p* value
Age, y.o, (range)	70 (46–85)	70 (42–90)	0.883
Male, n, (%)	100 (62.5)	65 (48.8)	0.024
Tumor diameter, mm, (range)	23 (8–53)	23 (7–89)	0.652
Tumor location, n, (%)			0.499
Head	115 (71.9)	88 (66.2)	
Body	35 (21.9)	37 (27.8)	
Tail	10 (6.2)	8 (6.0)	
Main pancreatic duct dilation, n, (%)	127 (79.3)	114 (85.7)	0.169
Obstructive jaundice, n, (%)	65 (40.6)	54 (40.6)	1.000

FNA, fine needle aspiration

### Outcomes of the endoscopic procedures

In the EUS-FNA group (N = 160), the median diameter of the long and short axes of the tumor was 23 mm (range, 7–53 mm) and 19 mm (5–40 mm), respectively. FNA was performed from the stomach in 57 patients, the duodenal bulb in 60 patients, the second portion of the duodenum in 39 patients, both the stomach and duodenal bulb in three patients, and the duodenal bulb and second portion in one patient. The size of the FNA needles was 19-gauge in 83 patients, 22-gauge in 46 patients, 25-gauge in 25 patients, 20-gauge in four patients, and 21-gauge in two patients. The median number of passes during FNA was 3 (range, 1–8) (Table 2). EUS-FNA had a cytological diagnostic sensitivity of 91.9% (147/160), a histological diagnostic sensitivity of 83.1% (133/160), and an overall diagnostic sensitivity for pancreatic cancer of 94.4% (151/160). Pathological samples were obtained during ERCP in 88 patients, pancreatic juice cytology in 24 patients, pancreatic ductal brush cytology in 23 patients, fluoroscopic pancreatic duct biopsy in nine patients, biliary juice cytology in 15 patients, bile duct brush cytology in 11 patients, and bile duct forceps biopsy in 48 patients. The diagnostic sensitivity of each method was as follows: pancreatic juice cytology: 25.0% (6/24: 95% confidence interval [CI] 0.11–0.44), pancreatic duct brush cytology: 34.8% (8/23: 95% CI 0.19–0.55), fluoroscopic pancreatic duct biopsy: 44.4% (4/9: 95% CI 0.19–0.73), bile juice cytology: 46.7% (7/15: 95% CI 0.25–0.70), bile duct brush cytology: 18.2% (2/11: 95% CI 0.051–0.48), and fluoroscopic bile duct biopsy: 41.7% (20/48: 95% CI 0.29–0.56). (Table 3) The diagnostic sensitivity of ERCP was 45.5% (40/88), and EUS-FNA showed a significantly higher diagnostic ability than ERCP (*p* < 0.001). In the EUS-FNA + ERCP combination group (n = 30), only one patient showed an additional effect of ERCP in eight patients who failed to obtain diagnosis with FNA. Adverse events were recognized in 1.3% (2/160) of patients who underwent EUS-FNA and in 10.2% (9/88) for those who underwent ERCP, with significantly lower occurrences in the former (*p* = 0.001). There was bleeding in one patient, abdominal pain in one patient in EUS-FNA, post-ERCP mild pancreatitis in five patients, post-endoscopic sphincterotomy bleeding in three patients, and bile duct perforation in one patient who underwent ERCP. All adverse events among those who underwent EUS-FNA were successfully managed conservatively, while two patients who underwent ERCP and experienced post-endoscopic sphincterotomy required hemostasis treatment (Table 4).

**Table 2:** Results of the EUS-FNA in patients with pancreatic cancer

	N = 160
Puncture routes, n, (%)	
Stomach	57 (35.6)
Duodenal bulb	60 (37.5)
2nd portion	39 (24.4)
Stomach/Duodenal bulb	3 (1.9)
Duodenal bulb/2nd portion	1 (0.6)
The size of the puncture needle, n, (%)	
19G	83 (51.9)
22G	46 (28.8)
25G	25 (15.6)
20G	4 (2.5)
21G	2 (1.2)
Number of punctures, median, (range)	3 (1–8)

FNA, fine needle aspiration

**Table 3:** Results of the diagnostic ability of EUS-FNA and ERCP in patients with pancreatic cancer

EUS-FNA	
Cytological diagnosis	91.9% (147/160)
Positive	125
Suspicious positive	22
Negative	13
Histological diagnosis	83.1% (133/160)
Adenocarcinoma	120
Suspicious adenocarcinoma	13
No diagnosis (atypia/inadequate/benign)	27 (18/8/1)
Overall	94.4% (151/160)
ERCP	
Sample collection methods	
Pancreatic juice cytology	25.0% (6/24)
Pancreatic ductal brush cytology	34.8% (8/23)
Fluoroscopic pancreatic duct biopsy	44.4% (4/9)
Biliary juice cytology	46.7% (7/15)
Bile duct brush cytology	18.2% (2/11)
Fluoroscopic bile duct biopsy	41.7% (20/48)
Overall	45.5% (40/88)

EUS-FNA, endoscopic ultrasound—fine needle aspiration; ERCP, endoscopic retrograde chorangiopancreatography

**Table 4:** Adverse events

	EUS-FNA (N = 160)	ERCP (N = 88)	*p* value
Adverse events, n (%)	2 (1.3)	9 (10.2)	0.001
Mild pancreatitis	0	5	
Bleeding	1	3	
Bile duct perforation	0	1	
Abdominal pain	1	0	

EUS-FNA, endoscopic ultrasonography—fine needle aspiration; ERCP, endoscopic retrograde chorangiopancreatography

### Surgical outcomes and prognosis

**Fig. 2:**
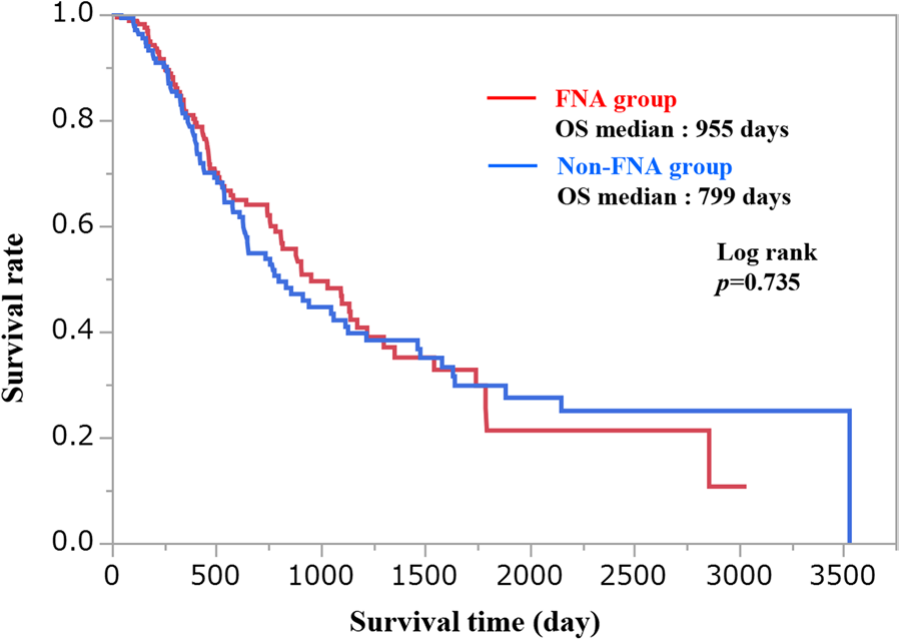
The overall survival analysis. FNA, fine needle aspiration

The following surgical procedures were performed: pancreatoduodenectomy (PD) in 182 patients, distal pancreatectomy (DP) in 75 patients, total pancreatectomy (TP) in 5 patients, middle pancreatecromy (MP) in one patient, and unresectable in 28 patients. The curability was as follows: R0, 190 patients; R1, 66 patients; R2, 37 patients. The final stage was as follows: I, 21 patients; II, 25 patients; III, 95 patients; IVa, 112 patients; IVb, 40 patients; and postoperative chemotherapy, 80.2% (235/293). There were no significant differences between the FNA and non-FNA groups in the factors that seemed to affect the prognosis of pancreatic cancer, such as surgical curability and adjuvant chemotherapy. In the prognostic study, a total of 256 patients had a curability of R0 or R1, and the recurrence rates were 54.3% (70/129) and 57.4% (73/127) in the FNA and non-FNA groups, respective. Furthermore, peritoneal dissemination occurred in 34.3% (24/70) in the FNA group and 21.9% (16/73) in the non-FNA group, neither of which showed significant differences (Table 5). The median survival times of the FNA and non-FNA groups were 955 days and 799 days, respectively, and there was no significant difference between the two groups (log-rank *p* = 0.735) (Fig. 2). Univariate and multivariate analyses showed that staging, curability, and adjuvant chemotherapy were independent risk factors for survival, but EUS-FNA was not (Table 6).

**Table 5:** The surgical curability and postoperative recurrence of two groups

	FNA group (N = 160)	Non-FNA group (N = 133)	*p* value
Curability, R0	104 (65.0)	86 (64.7)	1.000
Adjuvant Chemotherapy	134 (83.7)	101 (75.9)	0.106

	FNA group R0/R1(N = 129)	Non-FNA group R0/R1 (N = 127)	
Recurrence	70/129 (54.3)	73/127 (57.4)	0.616
Peritoneal dissemination	24/70 (34.3)	16/73 (21.9)	0.135

FNA, fine needle aspiration

**Table 6:** Cox proportional hazards model examining factors influencing prognosis

Factors		Univariate analysis		Multivariate analysis	
		HR (95% CI)	*p* value	HR (95% CI)	*p* value
Age	≧70 y.o	1.345 (0.974–1.858)	0.071	1.123 (0.789–1.596)	0.517
Sex	Male	1.385 (0.998–1.939)	0.050	1.222 (0.862–1.747)	0.260
Diagnostic method	EUS-FNA	0.946 (0.685–1.306)	0.735	0.882 (0.632–1.233)	0.464
Tumor location	Head	1.320 (0.924–1.924)	0.128	1.357 (0.943–1.994)	0.100
Tumor diameter	≧23 mm	1.531 (1.107–2.129)	0.009	1.359 (0.965–1.926)	0.078
Stage	IV	2.612 (1.865–3.690)	< 0.001	2.026 (1.407–2.942)	< 0.001
Curability	R0	0.383 (0.275–0.536)	< 0.001	0.463 (0.324–0.664)	< 0.001
Adjuvant Chemotherapy	Yes	0.561 (0.378–0.860)	0.009	0.446 (0.288–0.709)	< 0.001

EUS-FNA, endoscopic ultrasonography—fine needle aspiration; HR, hazard ratio; 95% CI, 95% confidence interval

## Discussion

In the present study, we analyzed the preoperative diagnosis of 293 patients with pancreatic cancer. The overall diagnostic sensitivity of EUS-FNA for pancreatic cancer was 94.4%, which was significantly higher than that of ERCP (45.5%) (*p* < 0.001). The adverse event rates associated with endoscopic procedures were significantly lower at 1.3% (2/160) in EUS-FNA than the 10.2% (9/88) in ERCP-related procedures (*p* = 0.001). There were no significant difference in the surgical outcomes, surgical curability, and recurrence rate between patients who underwent EUS-FNA (FNA group) and those who did not (non-FNA group). The Kaplan–Meier analysis also confirmed the absence of a significant difference in the overall survival between the two groups (*p* = 0.735). In the Cox proportional hazards model for overall survival, EUS-FNA was not an independent risk factor.

There are two endoscopic approaches for obtaining pathological specimens from pancreatic tumors: the transpapillary approach (ERCP) and the EUS-guided transintestinal approach (EUS-FNA). The transpapillary approach’s sensitivity for a malignant diagnosis is not very high, and endoscopic-related adverse events, such as post-ERCP pancreatitis, are also a concern [18]. On the other hand, EUS-FNA has a higher preoperative diagnostic capability than other modalities, with a diagnostic accuracy of 75–95% [19–24]. A previous study by Wakatsuki et al. compared the diagnostic ability of EUS-FNA (53 patients) with that of ERCP (30 patients) for pancreatic masses and reported that the sensitivity in the EUS-FNA and ERCP groups was 92.9% and 33.3% (*p* < 0.01), respectively. In our study, the overall sensitivity of EUS-FNA and ERCP-related procedures for pancreatic cancer was 94.4% and 45.5% (*p* < 0.001), respectively. These results suggest that EUS-FNA is a more sensitive preoperative diagnostic method for malignancy during the evaluation of pancreatic masses in comparison with ERCP-related procedures. In addition, recently, new needles with unique tip shapes have been utilized in fine needle biopsy (FNB) and have shown higher diagnostic yield with fewer needle passes [25–27]. Previously, rapid on-site evaluation (ROSE) has been considered to be effective in improve the diagnostic ability of EUS-FNA, but a recent study could not find any advantages in the diagnostic capability of ROSE during EUS-FNB for pancreatic cancer [28], although ROSE during ERCP-guided brushing for biliary strictures could still be an effective method [29].The overall incidence rate of FNA-related adverse events, such as bleeding, pancreatitis, and peritonitis, has been reported to be very low at 1–2%, and most could be managed conservatively [7, 30]. Considering its diagnostic ability and safety, EUS-FNA could be the first-line endoscopic procedure for the preoperative evaluation of suspected pancreatic cancer.

A few articles have reported the long-term outcomes of preoperative EUS-FNA in patients who underwent surgical resection of pancreatic cancer. A retrospective study by Ngamruengphong et al. evaluated 256 patients who underwent pancreatectomy, including 208 patients who underwent EUS-FNA for pancreatic tumors (FNA group) and 48 patients who did not undergo FNA (non-FNA group), with a median follow-up period of 23 months (range 0–111 months). They showed that gastric or peritoneal recurrence occurred in a total of 19 patients: 13 patients (7.7%) in the FNA group vs six patients (15.4%) in the non-FNA group (*p* = 0.21). In this study, three patients had a recurrence in the gastric wall: one (2.6%) patient in the non-FNA group and two patients (1.2%) in the FNA group (*p* = 0.46) [8]. Another retrospective study by Kudo et al. evaluated 82 patients with resectable pancreatic cancer. Of these, 54 underwent EUS-FNA before surgery (FNA group) and 28 underwent surgery without preoperative EUS-FNA (non-FNA group). The study reported that the median relapse-free survival (RFS) of the FNA and non-FNA groups was 742 and 265 days, respectively (*p* = 0.009), and the median overall survival (OS) was 1042 and 557 days, respectively (*p* = 0.007). The FNA group had better RFS and OS than the non-FNA group because more patients in the FNA group benefited from the chemotherapy administered immediately after surgery [9]. Tsutsumi et al. also performed a retrospective study to evaluate the impact of preoperative EUS-FNA. They divided 209 patients with pancreatic cancer into two groups: 126 patients who underwent preoperative EUS-FNA (FNA group) and 83 patients who did not (non-FNA group). They evaluated the long-term outcomes of preoperative EUS-FNA, especially disease-free survival, needle tract seeding, and recurrence. The Kaplan–Meier analysis indicated no significant difference in disease-free survival between the FNA and non-FNA groups. Furthermore, the site of recurrence was not significantly different between the two groups, and needle tract seeding was not observed [10]. In our study, the surgical curability (R0) was not significantly different between the FNA and non-FNA groups. There was no significant difference between the two groups in terms of recurrence rate, peritoneal dissemination incidence, and survival time after surgery. Moreover, in the multivariate analysis of the factors related to prognosis, staging, curability, and adjuvant chemotherapy were identified as dominant factors, but EUS-FNA itself was not. These studies, including ours, indicate that preoperative EUS-FNA does not adversely affect surgery or prognosis nor does it increase the risk of gastric wall or peritoneal recurrence in patients with resectable pancreatic cancer.

Recently, neoadjuvant chemotherapy for borderline resectable pancreatic cancer has been reported to have excellent effects on survival [31–35]. Motoi et al. also evaluated the efficacy of neoadjuvant chemotherapy for resectable pancreatic cancer. They performed a randomized controlled trial to compare neoadjuvant chemotherapy using gemcitabine and S-1 (NAC-GS) with upfront surgery (Up-S) for patients who underwent resection of pancreatic cancer. The NAC-GS group showed a significant increase in overall survival (Prep-02/JSAP05) [36]. Considering these results, the importance of preoperative pathological diagnosis might increase even in cases with indications for surgical resection. EUS-FNA is recommended as the first choice for tissue sample collection, considering its high diagnostic capability and lower adverse event rate than the transpapillary approach.

Although there was no evidence of the influence of preoperative EUS-FNA on the prognosis, needle tract seeding is a matter of concern. There have been several case reports of suspected tumor seeding related to preoperative EUS-FNA. Minaga et al*.* summarized the clinical features and outcomes of 15 cases of needle tract seeding. In 13 (86.7%) of 15 cases, EUS-FNA was performed via the gastric body to preferentially diagnose pancreatic body or tail lesions [37]. Yane et al. investigated the long-term outcomes, including the needle tract seeding ratio, of patients undergoing distal pancreatectomy for pancreatic body and tail cancer diagnosed preoperatively by EUS-FNA. Of the 301 patients analyzed, 176 underwent preoperative EUS-FNA (FNA group) and 125 did not (non-FNA group). Six patients (3.4%) in the FNA group were diagnosed with needle tract seeding, and the 5-year cumulative needle tract seeding rate estimated using Fine and Gray’s method was 3.8% (95% CI 1.6–7.8%)[15]. These data suggest that needle tract seeding after EUS-FNA has a non-negligible rate. In particular, since there is a possibility of needle tract seeding when performing EUS-FNA for a resectable tumor in the pancreatic body or tail, it is necessary to pay attention to the size of the needle and the number of punctures. Moreover, the possibility of needle tract seeding should always be considered in patients undergoing EUS-FNA for pancreatic cancer. Long-term follow-up with imaging studies may lead to the early detection of needle tract seeding and improve prognosis [38].

The present study has several limitations. A retrospective study design might have caused biases mainly in patient selection and treatment strategy. ERCP related procedures were more frequently used in the former part of the study, whereas EUS-FNA was more frequently used in the latter part, which might have also caused bias in the clinical outcomes. Since the study included only high-volume centres for both EUS- and ERCP-related procedures, the external validity might be poor, especially for the endoscopic procedure.

In conclusion, EUS-FNA was safe and has a high diagnostic ability during the preoperative examination for pancreatic cancer. It was considered the first choice without the influence of surgical curability, postoperative recurrence, peritoneal dissemination, and prognosis. However, since needle tract seeding is observed to have a certain probability, EUS-FNA for resectable tumors, especially those located in the pancreatic body or tail, requires careful consideration of the relationship between risk and benefit. A randomized controlled trial with a multicenter setting is needed to confirm the study results.

## Data Availability

The data that support the findings of this study are available from the corresponding author upon reasonable request.

## Abbreviations

EUS-FNA: Endoscopic ultrasound–guided fine needle aspiration
FNB: Fine needle biopsy
ERCP: Endoscopic retrograde cholangiopancreatography
UMIN: University hospital medical information network
95% CI: 95% Confidence interval
EST: Endoscopic sphincterotomy
PD: Pancreatoduodenectomy
DP: Distal pancreatectomy
TP: Total pancreatectomy
MP: Middle pancreatecromy
RFS: Relapse-free survival
OS: Overall survival
